# 1,3-Bis{[5-(pyridin-2-yl)-1,3,4-oxadiazol-2-yl]sulfan­yl}propan-2-one

**DOI:** 10.1107/S1600536811001140

**Published:** 2011-01-15

**Authors:** Chao-Hui Xia, Chun-Bo Mao, Ben-Lai Wu

**Affiliations:** a Department of Chemistry, Zhengzhou University, Zhengzhou 450052, People’s Republic of China; b Henan Vocational College of Chemical Technology, Zhengzhou 450052, People’s Republic of China

## Abstract

In the distorted W-shaped mol­ecule of the title compound, C_17_H_12_N_6_O_3_S_2_, a twofold axis passes through the carbonyl group. The mol­ecules stack in the crystal through π–π inter­actions [centroid—centroid distance = 3.883 Å] and weak C—H⋯N hydrogen-bonding inter­actions, forming a three-dimensional architecture.

## Related literature

For the use of oxadiazole-containing compounds with symmetrical or asymmetrical structures in coordination chemistry, see: Du *et al.* (2006 ▶); Fang *et al.* (2002 ▶); Wu *et al.* (2010 ▶); Ye *et al.* (2007 ▶). For a similar propanone-bridged dithio­ether compound, see: Wu *et al.* (2005 ▶).
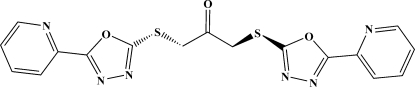


## Experimental

### 

#### Crystal data

C_17_H_12_N_6_O_3_S_2_
*M* *_r_* = 412.45
Monoclinic, 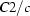

*a* = 14.266 (3) Å
*b* = 7.8342 (16) Å
*c* = 16.703 (3) Å
β = 100.25 (3)°
*V* = 1837.0 (6) Å^3^
*Z* = 4
Mo *K*α radiation
μ = 0.32 mm^−1^
*T* = 293 K
0.20 × 0.20 × 0.20 mm

#### Data collection

Siemens SMART CCD diffractometer
Absorption correction: multi-scan (*SADABS*; Sheldrick, 1996 ▶) *T*_min_ = 0.913, *T*_max_ = 1.000
8929 measured reflections
1607 independent reflections
1448 reflections with *I* > 2σ(*I*)
*R*_int_ = 0.050

#### Refinement

*R*[*F* ^2^ > 2σ(*F* ^2^)] = 0.076
*wR*(*F* ^2^) = 0.134
*S* = 1.24
1607 reflections
128 parameters
H-atom parameters constrained
Δρ_max_ = 0.33 e Å^−3^
Δρ_min_ = −0.21 e Å^−3^

### 

Data collection: *SMART* (Siemens, 1996 ▶); cell refinement: *SAINT* (Siemens, 1994 ▶); data reduction: *SAINT*; program(s) used to solve structure: *SHELXS97* (Sheldrick, 2008 ▶); program(s) used to refine structure: *SHELXL97* (Sheldrick, 2008 ▶); molecular graphics: *SHELXTL* (Sheldrick, 2008 ▶); software used to prepare material for publication: *SHELXL97*.

## Supplementary Material

Crystal structure: contains datablocks I, global. DOI: 10.1107/S1600536811001140/hg2787sup1.cif

Structure factors: contains datablocks I. DOI: 10.1107/S1600536811001140/hg2787Isup2.hkl

Additional supplementary materials:  crystallographic information; 3D view; checkCIF report

## Figures and Tables

**Table 1:** Hydrogen-bond geometry (Å, °)

*D*—H⋯*A*	*D*—H	H⋯*A*	*D*⋯*A*	*D*—H⋯*A*
C2—H2*B*⋯N3^i^	0.99	2.51	3.388 (4)	148
C9—H9⋯N1^ii^	0.95	2.54	3.449 (5)	161

Symmetry codes: (i) 
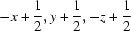
; (ii) 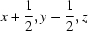.

**Fig. 1.**
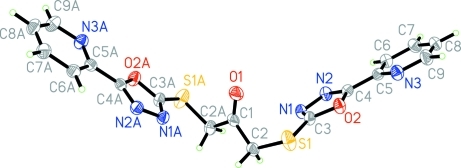
Perspective view of (I) with atom numbering scheme. Atom displacement ellipsoids are shown at 30% probability level. Symmetry codes, as in Table 1.

**Fig. 2.**
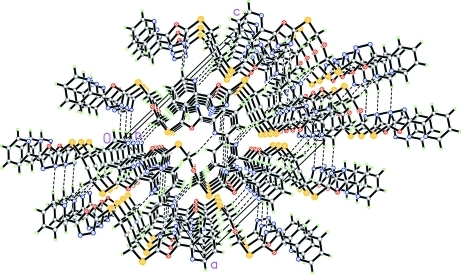
Packing diagram of (I), showing intermolecular π–π interactions and weaker C—H···N hydrogen-bonding interactions.

## supplementary crystallographic information

### Comment

1,3,4-Oxadiazole and its derivatives exhibits good luminous properties, biological activity, and as well as flexibility in crystal engineering. So far, a great deal of oxadiazole-containing compounds with symmetrical or asymmetrical structures has been prepared and especially used in coordination chemistry (Du *et al.*, 2006; Fang, *et al.*, 2002; Ye, *et al.*, 2007; Wu, *et al.*, 2010). We herein describe the structure of the title compound, (I), which is a new oxadiazole-containing thioethers ligand with a propan-2-one moiety at the linkage.

In (I), there is a twofold rotation axis passing through the C1—O1 carbonyl group of the propanone moiety (Fig. 1). Two 5-(pyridin-2-yl)-1,3,4-oxadiazole-2-thio groups are oriented anti to bind the propanone moiety, and thus form the compound (I) with a distorted "W-shaped" configuration, being similar to another propanone-bridged dithioether compound ( Wu, *et al.*, 2005). The S1 atom deviates from the least-squares plane of C2/C2^i^/C1/O1 by 0.387 (7) Å [(i) 1 - *x*, *y*, -*z* + 1/2]. The dihedral angle of pyridin-2-yl and oxadiazole group is 2.8 (1)°, indicating that pyridin-2-yl and oxadiazole group are almost coplanar in 5-(pyridin-2-yl)-1,3,4-oxadiazole-2-thio group.The dihedral angle of two 5-(pyridin-2-yl)-1,3,4-oxadiazole-2-thio is 68.8 (1)°, while that of the propanone moiety and the pyrimidine ring is 70.7 (1)°. Notably, the molecules stack in the crystal lattice through intermolecular π–π interactions of pyridyl and oxadiazole groups with center-to-center distance of 3.883 Å and weaker C—H···N hydrogen-bonding interactions to form a three-dimensional architecture (Fig. 2).

### Experimental

Sodium methylate (0.540 g, 10 mmol) and 5-(2-pyridyl)-2-mercapto-1,3,4- oxadiazole (1.79 g, 10 mmol) were vigorously stirred in MeOH (50 ml) for 1 h, before quantitative 1,3-dichloro-2-propanone (0.635 g, 5 mmol) was added. The resulting solution was heated at 373 K for 12 h and then filtered after cooled to room temperature. Removal of the solvent from the yellow filtrate created yellow powder which was washed with water and recrystallized from methanol to produce yellow crystals of (I) (yield 2.572 g, 64%; m.p. 174–175 °C). Slow evaporation of methanol solution of (I) for two weeks created block yellow crystals suitable for X-ray diffraction.

### Refinement

All H atoms were positioned geometrically and constrained to ride on their parent atoms.

### Crystal data

**Table:**

C_17_H_12_N_6_O_3_S_2_	*F*(000) = 848
*M**_r_* = 412.45	*D*_x_ = 1.491 Mg m^−^^3^
Monoclinic, *C*2/*c*	Mo *K*α radiation, λ = 0.71073 Å
Hall symbol: -C 2yc	Cell parameters from 2643 reflections
*a* = 14.266 (3) Å	θ = 2.5–29.1°
*b* = 7.8342 (16) Å	µ = 0.32 mm^−^^1^
*c* = 16.703 (3) Å	*T* = 293 K
β = 100.25 (3)°	Block, yellow
*V* = 1837.0 (6) Å^3^	0.20 × 0.20 × 0.20 mm
*Z* = 4	

### Data collection

**Table:**

Siemens SMART CCD diffractometer	1607 independent reflections
Radiation source: fine-focus sealed tube	1448 reflections with *I* > 2σ(*I*)
graphite	*R*_int_ = 0.050
ω scans	θ_max_ = 25.0°, θ_min_ = 2.5°
Absorption correction: multi-scan (*SADABS*; Sheldrick, 1996)	*h* = −16→16
*T*_min_ = 0.913, *T*_max_ = 1.000	*k* = −9→9
8929 measured reflections	*l* = −19→19

### Refinement

**Table:**

Refinement on *F*^2^	Primary atom site location: structure-invariant direct methods
Least-squares matrix: full	Secondary atom site location: difference Fourier map
*R*[*F*^2^ > 2σ(*F*^2^)] = 0.076	Hydrogen site location: inferred from neighbouring sites
*wR*(*F*^2^) = 0.134	H-atom parameters constrained
*S* = 1.24	*w* = 1/[σ^2^(*F*_o_^2^) + (0.0357*P*)^2^ + 3.0521*P*] where *P* = (*F*_o_^2^ + 2*F*_c_^2^)/3
1607 reflections	(Δ/σ)_max_ < 0.001
128 parameters	Δρ_max_ = 0.33 e Å^−^^3^
0 restraints	Δρ_min_ = −0.21 e Å^−^^3^

### Special details

**Table:**

Geometry. All e.s.d.'s (except the e.s.d. in the dihedral angle between two l.s. planes) are estimated using the full covariance matrix. The cell e.s.d.'s are taken into account individually in the estimation of e.s.d.'s in distances, angles and torsion angles; correlations between e.s.d.'s in cell parameters are only used when they are defined by crystal symmetry. An approximate (isotropic) treatment of cell e.s.d.'s is used for estimating e.s.d.'s involving l.s. planes.
Refinement. Refinement of *F*^2^ against ALL reflections. The weighted *R*-factor *wR* and goodness of fit *S* are based on *F*^2^, conventional *R*-factors *R* are based on *F*, with *F* set to zero for negative *F*^2^. The threshold expression of *F*^2^ > σ(*F*^2^) is used only for calculating *R*-factors(gt) *etc*. and is not relevant to the choice of reflections for refinement. *R*-factors based on *F*^2^ are statistically about twice as large as those based on *F*, and *R*- factors based on ALL data will be even larger.

### Fractional atomic coordinates and isotropic or equivalent isotropic displacement parameters (Å^2^)

**Table:**

	*x*	*y*	*z*	*U*_iso_*/*U*_eq_	
S1	0.19304 (8)	0.68102 (19)	0.24214 (6)	0.0785 (5)	
O1	0.0000	0.5351 (5)	0.2500	0.0640 (11)	
O2	0.28820 (16)	0.5281 (3)	0.37180 (14)	0.0525 (7)	
N1	0.1587 (2)	0.6557 (4)	0.39768 (17)	0.0485 (8)	
N2	0.2056 (2)	0.5752 (4)	0.46924 (17)	0.0488 (8)	
N3	0.4202 (2)	0.3330 (4)	0.46674 (18)	0.0549 (8)	
C1	0.0000	0.6876 (7)	0.2500	0.0453 (12)	
C2	0.0834 (2)	0.7944 (5)	0.2358 (2)	0.0531 (10)	
H2A	0.0928	0.8879	0.2763	0.064*	
H2B	0.0675	0.8471	0.1812	0.064*	
C3	0.2100 (2)	0.6238 (5)	0.3439 (2)	0.0472 (9)	
C4	0.2798 (2)	0.5024 (4)	0.45132 (19)	0.0394 (8)	
C5	0.3528 (2)	0.4016 (4)	0.5029 (2)	0.0431 (8)	
C6	0.3488 (3)	0.3797 (5)	0.5839 (2)	0.0500 (9)	
H6	0.2993	0.4304	0.6070	0.060*	
C7	0.4183 (3)	0.2824 (5)	0.6308 (2)	0.0587 (10)	
H7	0.4172	0.2635	0.6868	0.070*	
C8	0.4890 (3)	0.2137 (5)	0.5952 (3)	0.0610 (11)	
H8	0.5382	0.1475	0.6263	0.073*	
C9	0.4878 (3)	0.2419 (5)	0.5144 (3)	0.0637 (11)	
H9	0.5375	0.1942	0.4905	0.076*	

### Atomic displacement parameters (Å^2^)

**Table:**

	*U*^11^	*U*^22^	*U*^33^	*U*^12^	*U*^13^	*U*^23^
S1	0.0624 (7)	0.1289 (11)	0.0478 (6)	0.0273 (7)	0.0196 (5)	0.0245 (7)
O1	0.076 (3)	0.053 (3)	0.056 (2)	0.000	−0.0065 (19)	0.000
O2	0.0435 (14)	0.0728 (18)	0.0427 (14)	0.0122 (12)	0.0115 (11)	0.0052 (12)
N1	0.0445 (17)	0.059 (2)	0.0407 (16)	0.0085 (14)	0.0045 (13)	−0.0009 (14)
N2	0.0510 (18)	0.058 (2)	0.0365 (16)	0.0064 (15)	0.0064 (13)	−0.0037 (14)
N3	0.0491 (18)	0.066 (2)	0.0516 (18)	0.0100 (16)	0.0151 (15)	0.0038 (16)
C1	0.060 (3)	0.047 (3)	0.024 (2)	0.000	−0.006 (2)	0.000
C2	0.052 (2)	0.064 (3)	0.041 (2)	0.0022 (19)	0.0029 (16)	0.0107 (18)
C3	0.0380 (19)	0.059 (2)	0.044 (2)	0.0037 (17)	0.0061 (16)	0.0031 (18)
C4	0.0417 (19)	0.044 (2)	0.0327 (17)	−0.0036 (15)	0.0077 (14)	−0.0048 (15)
C5	0.0433 (19)	0.042 (2)	0.0431 (19)	−0.0034 (16)	0.0068 (16)	−0.0051 (16)
C6	0.054 (2)	0.054 (2)	0.042 (2)	0.0065 (18)	0.0084 (17)	−0.0068 (18)
C7	0.067 (3)	0.060 (3)	0.047 (2)	0.005 (2)	0.0047 (19)	0.0036 (19)
C8	0.054 (2)	0.058 (3)	0.067 (3)	0.006 (2)	0.000 (2)	0.017 (2)
C9	0.049 (2)	0.070 (3)	0.074 (3)	0.015 (2)	0.017 (2)	0.012 (2)

### Geometric parameters (Å, °)

**Table:**

S1—C3	1.732 (4)	C2—H2A	0.9900
S1—C2	1.785 (4)	C2—H2B	0.9900
O1—C1	1.195 (6)	C4—C5	1.460 (5)
O2—C3	1.356 (4)	C5—C6	1.374 (5)
O2—C4	1.369 (4)	C6—C7	1.379 (5)
N1—C3	1.281 (4)	C6—H6	0.9500
N1—N2	1.411 (4)	C7—C8	1.369 (5)
N2—C4	1.283 (4)	C7—H7	0.9500
N3—C5	1.336 (4)	C8—C9	1.365 (6)
N3—C9	1.341 (5)	C8—H8	0.9500
C1—C2	1.507 (5)	C9—H9	0.9500
C1—C2^i^	1.507 (5)		
			
C3—S1—C2	98.92 (17)	N2—C4—C5	129.3 (3)
C3—O2—C4	101.9 (2)	O2—C4—C5	118.4 (3)
C3—N1—N2	105.2 (3)	N3—C5—C6	123.6 (3)
C4—N2—N1	106.7 (3)	N3—C5—C4	116.5 (3)
C5—N3—C9	116.4 (3)	C6—C5—C4	119.9 (3)
O1—C1—C2	123.7 (2)	C5—C6—C7	118.5 (3)
O1—C1—C2^i^	123.7 (2)	C5—C6—H6	120.8
C2—C1—C2^i^	112.6 (5)	C7—C6—H6	120.8
C1—C2—S1	115.0 (3)	C8—C7—C6	118.8 (4)
C1—C2—H2A	108.5	C8—C7—H7	120.6
S1—C2—H2A	108.5	C6—C7—H7	120.6
C1—C2—H2B	108.5	C9—C8—C7	119.0 (4)
S1—C2—H2B	108.5	C9—C8—H8	120.5
H2A—C2—H2B	107.5	C7—C8—H8	120.5
N1—C3—O2	113.9 (3)	N3—C9—C8	123.7 (4)
N1—C3—S1	129.8 (3)	N3—C9—H9	118.2
O2—C3—S1	116.3 (2)	C8—C9—H9	118.2
N2—C4—O2	112.3 (3)		
			
C3—N1—N2—C4	−0.3 (4)	C3—O2—C4—C5	179.9 (3)
O1—C1—C2—S1	−13.9 (3)	C9—N3—C5—C6	−1.6 (5)
C2^i^—C1—C2—S1	166.1 (3)	C9—N3—C5—C4	179.3 (3)
C3—S1—C2—C1	−64.4 (3)	N2—C4—C5—N3	176.6 (3)
N2—N1—C3—O2	0.1 (4)	O2—C4—C5—N3	−3.8 (5)
N2—N1—C3—S1	178.8 (3)	N2—C4—C5—C6	−2.5 (6)
C4—O2—C3—N1	0.2 (4)	O2—C4—C5—C6	177.1 (3)
C4—O2—C3—S1	−178.8 (2)	N3—C5—C6—C7	0.4 (6)
C2—S1—C3—N1	−0.5 (4)	C4—C5—C6—C7	179.4 (3)
C2—S1—C3—O2	178.3 (3)	C5—C6—C7—C8	0.9 (6)
N1—N2—C4—O2	0.5 (4)	C6—C7—C8—C9	−0.8 (6)
N1—N2—C4—C5	−179.8 (3)	C5—N3—C9—C8	1.7 (6)
C3—O2—C4—N2	−0.4 (4)	C7—C8—C9—N3	−0.5 (7)

Symmetry codes: (i) −*x*, *y*, −*z*+1/2.

### Hydrogen-bond geometry (Å, °)

**Table:**

*D*—H···*A*	*D*—H	H···*A*	*D*···*A*	*D*—H···*A*
C2—H2B···N3^ii^	0.99	2.51	3.388 (4)	148
C9—H9···N1^iii^	0.95	2.54	3.449 (5)	161

Symmetry codes: (ii) −*x*+1/2, *y*+1/2, −*z*+1/2; (iii) *x*+1/2, *y*−1/2, *z*.
